# Therapeutic potential of functionalized siRNA nanoparticles on regression of liver cancer in experimental mice

**DOI:** 10.1038/s41598-019-52142-4

**Published:** 2019-11-01

**Authors:** Azmat Ali Khan, Amer M. Alanazi, Mumtaz Jabeen, Arun Chauhan, Mohammad Azam Ansari

**Affiliations:** 1 Pharmaceutical Biotechnology Laboratory, Department of Pharmaceutical Chemistry, College of Pharmacy, King Saud University, Riyadh, 11451 Saudi Arabia; 2 Section of Genetics, Department of Zoology, Aligarh Muslim University, Aligarh, India; 3 Department of Neuroimmunology, School of Health and Medicine, University of North Dakota, Grand Forks, ND USA; 4 Department of Epidemic Disease Research, Institutes of Research and Medical Consultations (IRMC), Imam Abdulrahman Bin Faisal University, 31441 Dammam, Saudi Arabia

**Keywords:** Nanoparticles, Drug development

## Abstract

Short interfering RNA (siRNA) possesses special ability of silencing specific gene. To increase siRNA stability, transportation and its uptake by tumor cells, effective delivery to the appropriate target cells is a major challenge of siRNA-based therapy. In the present study, an effective, safe and biocompatible survivin siRNA encapsulated, GalNAc decorated PEGylated PLGA nanoconjugates (NCs) viz., GalNAc@PEG@*siRNA*-PLGA were engineered and their synergistic antitumor efficacy was evaluated for targeted delivery in HCC bearing experimental mice. GalNAc@PEG@*siRNA*-PLGA NCs were characterized for size, bioavailability, toxicity and biocompatibility. Their antitumor potential was evaluated considering gene silencing, apoptosis, histopathology and survival of treated mice. Exceptional accumulation of hepatocytes, reduction in survivin expression and prominent regression in tumor size confirmed the ASGPR-mediated uptake of ligand-anchored NCs and silencing of survivin gene in a targeted manner. Increased DNA fragmentation and potential modulation of caspase-3, Bax and Bcl-2 factors specified the induction of apoptosis that helped in significant inhibition of HCC progression. The potential synchronous and tumor selective delivery of versatile NCs indicated the effective payloads towards the target site, increased apoptosis in cancer cells and improved survival of treated animals.

## Introduction

Hepatocellular carcinoma (HCC) is one of the primary liver cancers displaying high frequency of relapse and metastasis^1^. It is ranked as the third leading cause of mortality and the fifth most common type of malignancy in the world causing about 500,000 deaths annually^2,3^. Despite causing overall side-effects and drug resistance, chemotherapy still persists as a main line of treatment for HCC. Many other therapeutic approaches are nowadays being explored for effective treatment of different types of cancers. Among these methods, siRNA induced RNAi represents an effective and powerful strategy to silence a wide range of cancer-associated genes^4^. However, therapeutic activity of naked siRNA faces many challenges inside the host body. Due to short half-life, easy degradation in serum and rapid renal clearance^5^, siRNA is unable to reach the target site in optimum quantity. At cellular level also, negatively charged siRNA is repulsed from the same charged cell membrane thereby resulting in poor cellular uptake. Therefore, to improve the pharmacokinetics and stability of siRNA *in vivo*, advanced strategies are needed for its delivery to the target site.

Nanoparticle-based therapeutics has the ability to selectively deliver the bio-therapeutic agents to the cytoplasm of cancer cells. Various studies have shown the potential of suitable carriers for successful drug delivery to the target site through the biological barriers^6–9^. Successful delivery of siRNA to the tumor cells using nano-carriers appears to be a promising nanomedicine approach for superior cancer therapy^10,11^. The polylactic acid-co-glycolic acids (PLGAs) are nanoparticles approved by the Food and Drug Administration (FDA) for clinical use since 1969 and are been widely used in pharmaceutics. Due to their high stability, high loading efficiency, sustained release, biodegradability and cellular uptake by endocytosis; they are vastly employed for chemotherapeutic delivery of anticancer drug or siRNA^12,13^. Large amount of endosomal escape and release from PLGA’s results in high efficiency of encapsulated molecule^14,15^. The coupling of PLGA with polyethylene glycol (PEG) polymer is one of the most proficient modifications of all. Similarly to PLGA, usage of PEG in therapeutic devices is also approved by FDA and by the European Medicine Agency as well^16^. PLGAs decorated with PEG layer show an increase in half-life, increased circulation in blood, and the ability to disguise itself from recognition by the mononuclear phagocytic system^17^. Still, the nanoparticles show reduced delivery of drug to tumor site because of non-specific distribution and drug release to other healthy organs. Therefore, to achieve maximum therapeutic efficacy of nano-delivery systems and modulation of drug release, potential functionalization of nanoparticles is further needed for selective targeting of the drug.

Specific targeted delivery can be interpreted as an important strategy for HCC therapy that can be achieved by targeting the overexpressed surface receptors on hepatocytes^18^. Asialoglycoprotein receptor (ASGPR) is a Ca^2+^ dependent human C-type lectin transmembrane receptor that is expressed in high density on the surface of hepatocytes and hepatic cancer cells and minimally present elsewhere in the body. Attributes like easy approach from vascular compartment, rapid internalization of large molecules and high affinity makes it an ideal target for hepatocyte-specific targeting^19–21^. The main advantage of ASGPR is its affinity towards various ligands with as simple as carbohydrates^22,23^. In view of this aspect, among various ligands, N-acetylgalactosamine (GalNAc), an oligosaccharide, has high affinity towards ASGPRs and decoration of nanoparticles with GalNAc will be of great importance in selective delivery of gene of interest to HCC tissue via ASGPR receptors.

Taking into consideration the above mentioned attributes of a suitable nanoparticle, we envisaged the synthesis of multi-functional NCs. In this report, survivin siRNA was encapsulated in biodegradable PLGA nanoparticles that were PEGylated and then functionalized with GalNAc ligand to target experimental HCC. The designed NCs (GalNAc@PEG@*siRNA*-PLGA) were expected to specifically bind ASGPR receptors of HCC cells, show high transfection efficiency resulting in potential knockdown of survivin gene.

## Results

### Chemical structure of GalNAc@PEG@siRNA-PLGA NC

**Figure 1:**
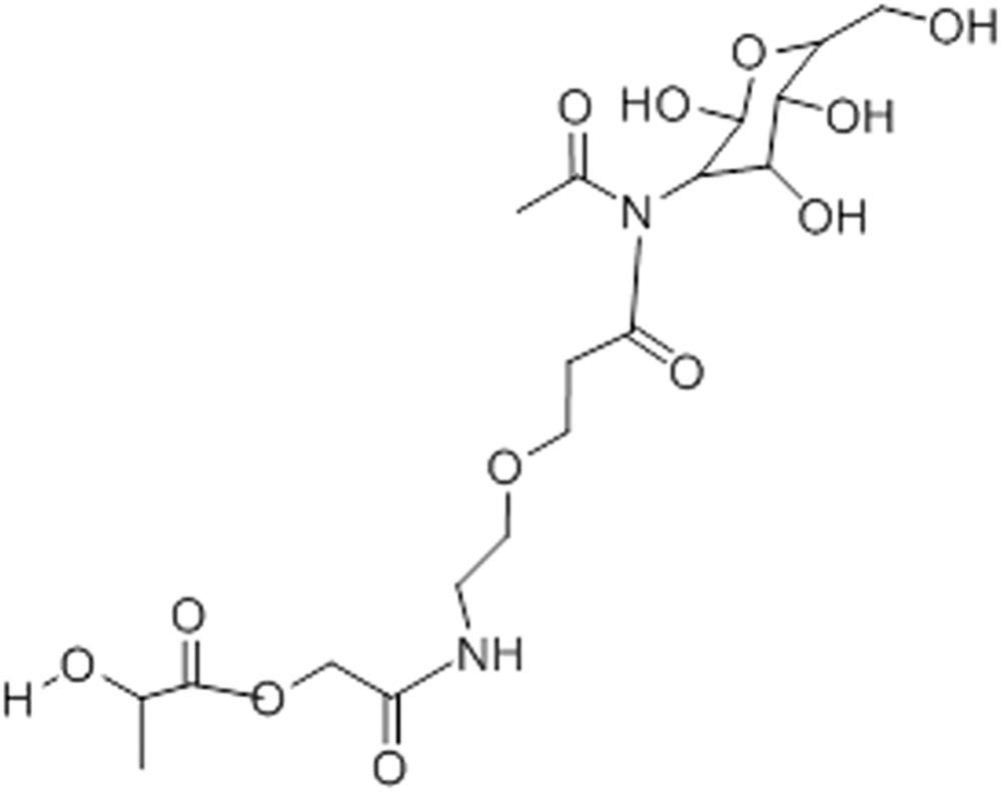
Chemical Structure of GalNAc@PEG@siRNA-PLGA nanoconjugate.

The siRNA loaded PLGA nanoparticles were prepared by the double emulsion method. The carboxyl groups present on surface of PLGA of *siRNA*-PLGA nanoparticles were increased by adding polyethylene-maleic anhydride (PEMA). Now, these modified PEMA treated *siRNA*-PLGA were incubated with N-hydroxysuccinimide (NHS) and 1-(3-dimethylaminopropyl)-3-ethylcarbodimide hydrochloride (EDC). The resulting NHS-activated *siRNA*-PLGA was covalently linked to NH-PEG-COOH. The resulting *siRNA*-PLGA-PEG-COOH nanoparticles were again activated using NHS. The GalNAc was now covalently linked to PEG-decorated *siRNA*-PLGA resulting in the formation of GalNAc@PEG@*siRNA*-PLGA nanoconjugates (NCs) (Fig. 1).

### Characterization of GalNAc@PEG@*siRNA*-PLGA

**Figure 2:**
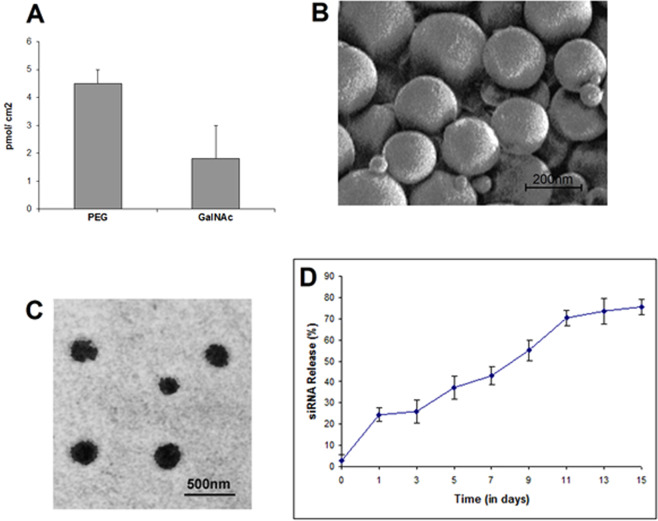
Density, size, surface morphology and release profile of GalNAc@PEG@*siRNA*-PLGA. (**A)** The density of PEG and GalNAc on the surface of *siRNA-*PLGA and PEG@*siRNA*-PLGA, respectively was estimated after each reaction step. The total surface area was calculated according to normal Gaussian particle size distribution. **(B)** SEM image of GalNAc@PEG@*siRNA*-PLGA showing shape and surface morphology **(C)** TEM image showing the size and surface morphology of GalNAc@PEG@*siRNA*-PLGA NCs. **(D)** *In vitro* release pattern of siRNA from NC upon dispersion in PBS (pH 7.4) demonstrates 75% release till 15^th^ day. Data are the mean ± SD of three sets of independent experiments.

The conjugation chemistry of PEG and GalNAc was indirectly scored from the amount of unreacted PEG and GalNAc after complete synthesis of NC. Proper measures were taken to assure no loss of molecules through non-specific adsorption. Figure 2A shows the density of PEG (4.5 ± 0.5 pmol/cm^2^) and GalNAc (1.8 ± 0.06 pmol/cm^2^) attached onto the surface of *siRNA*-PLGA and PEG@*siRNA*-PLGA, respectively. These values are in accordance with other published report^26^.

Size, shape and surface morphology was examined via SEM and TEM (Fig. 2B,2C respectively). Synthesized siRNA encapsulated PLGA nanoparticle had an average size of approximately 190 ± 20 nm. After PEGylation and conjugation, GalNAc@PEG@*siRNA*-PLGA NC appeared to be approximately 210 nm in size with rough surface and spherical shape. PDI and zeta-potential values of in-house developed NC were 0.108 ± 0.016 and −6.7 ± 0.4 mV, respectively. The % entrapment efficiency of GalNAc@PEG@*siRNA*-PLGA was 53 ± 0.5% with siRNA load of 690 ± 28 ng/mg NC.

The release kinetics of siRNA from GalNAc@PEG@*siRNA*-PLGA was analysed in PBS (pH 7.4) at 37 °C. Initially, there was approx. 25% release of encapsulated siRNA in quick burst within 24 h (Fig. 2D). Following an interval of two days, siRNA was again released in a slow and sustained manner. Till 15^th^ day, almost 75% of the total siRNA was released from the matrix.

### Specific targeting of GalNAc@PEG@*siRNA*-PLGA to HCC cells

**Figure 3:**
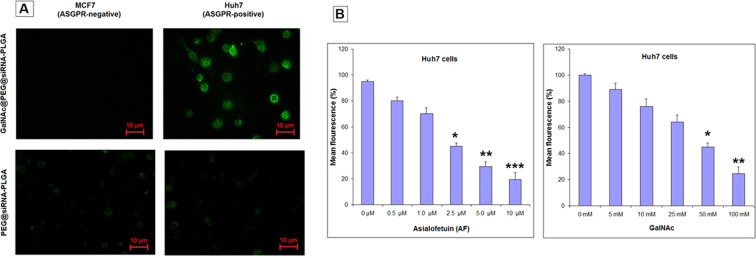
Specific targeting of GalNAc@PEG@*siRNA*-PLGA to HCC cells. (**A)** Huh7 and MCF7 cells were incubated with fluorescently-labeled PEG@*siRNA*-PLGA or fluorescently-labeled GalNAc@PEG@*siRNA*-PLGA. Green color of Alexa-488 was observed on the cell surface by a fluorescence microscope. **(B)** Competitive inhibition of GalNAc@PEG@*siRNA*-PLGA NCs into Huh7 cells upon pre-incubation with increasing concentrations of AF **(a)** and GalNAc **(b)** molecules. Data are the means ± SD of three sets of different experiments. *P < 0.05, **P < 0.01, ***P < 0.001 versus control.

Under a fluorescent microscope, localization of NCs to the cell periphery was observed after 30 min of incubation (Fig. 3A). Specific targeting by GalNAc@PEG@*siRNA*-PLGA was observed only in ASGPR-positive Huh7 cells and not in ASGPR-negative MCF7 cells. The presence of GalNAc resulted in rapid targeting of NCs towards ASGPR receptors as vizualized with ~2.5-fold increase in fluorescence intensity. To confirm that targeting is due to GalNAc and not to some phenotypic differences between the two cell lines, Huh7 and MCF7 cells were incubated with PEG@*siRNA*-PLGA where GalNAc was not present. The PEG@*siRNA*-PLGA formulation targeted both types of cells irrespective of being ASGPR-positive/negative. However, extent of incorporation was lesser than NCs.

To re-evaluate the evidence of specific binding of NC to ASGPR-positive Huh7 cells, Asialofetuin (AF) and GalNAc were employed as competitive inhibitors. Cells were treated with increasing concentrations of AF or GalNAc prior to the treatment of GalNAc@PEG@*siRNA*-PLGA NC. A dose-dependent binding inhibition was observed where increase in AF or GalNAc concentration resulted in significant decrease in fluorescence intensity (Fig. 3B). Almost 80% of NC binding to Huh7 cells was inhibited (P < 0.01) in the presence of 10 µM AF (Fig. 3B, left), while 75% (P < 0.01) inhibition was observed in the presence of 100 mM GalNAc (Fig. 3B, right).

### Assessment of toxicity levels of GalNAc@PEG@*siRNA*-PLGA

#### Hemolysis testing

**Figure 4:**
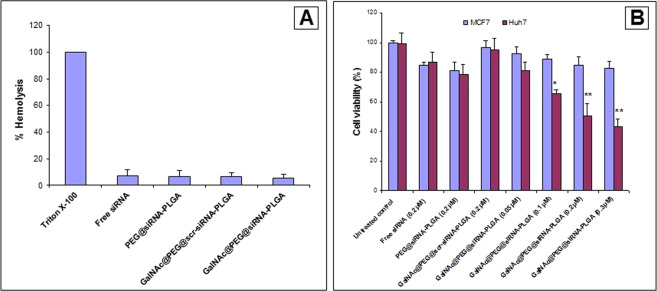
*In vitro* toxicity levels of GalNAc@PEG@*siRNA*-PLGA. (**A**) The extent of hemolysis caused by NCs was measured by erythrocyte lysis test. Graph shows the hemolysis of human erythrocytes after incubation with free siRNA, PEG@*siRNA*-PLGA, GalNAc@PEG@scr-*siRNA*-PLGA and GalNAc@PEG@*siRNA*-PLGA. **(B)** MTT assay for evaluating cytotoxic effect of NC to Huh7 cells. The cells were treated with free siRNA (0.2 µM), PEG@*siRNA*-PLGA (0.2 µM) and GalNAc@PEG@*siRNA*-PLGA (0.05 µM, 0.1 µM, 0.2 µM and 0.3 µM). Untreated cells were taken as a control. Data are the means ± SD of three sets of different experiments. *P < 0.05, **P < 0.01 versus untreated control.

Lysis of 100% red blood cells (RBCs) by Triton X-100 was taken as a positive control. As shown in Fig. 4A, GalNAc@PEG@*siRNA*-PLGA induced negligible RBC lysis. The intrinsic lytic ability of free siRNA, PEG@*siRNA*-PLGA and GalNAc@PEG@*scr-siRNA*-PLGA were also examined. siRNA in different formulations was negligibly toxic to RBCs.

#### *In vivo* toxicity

Levels of toxicity of NC *in vivo* were examined by evaluating the renal and hepatic function parameters in sera of tested healthy mice. After treatment with GalNAc@PEG@*siRNA*-PLGA, sera of mice were assayed for creatinine and ALP, AST and ALT levels. As shown in Table 1, sera enzyme levels were very similar to those of the healthy control group. The NC along with free siRNA, PEG@*siRNA*-PLGA and GalNAc@PEG@*scr-siRNA*-PLGA induced neither hepatic nor renal toxicities.

**Table 1:** Effect of GalNAc@PEG@*siRNA*-PLGA on *in vivo* toxicity.

Treatment Groups	Creatinine (mg%)		ALT (U/I)		Total Bilirubin	
	Day 0	Day 6	Day 0	Day 6	Day 0	Day 6
Healthy (positive) control	0.24 ± 0.01	0.21 ± 0.05	37.56 ± 0.04	36.39 ± 0.05	0.185 ± 0.02	0.192 ± 0.03
Untreated (negative) control	0.32 ± 0.04	0.34 ± 0.05	42.32 ± 0.02	43.76 ± 0.07	0.318 ± 0.05	0.343 ± 0.07
Free siRNA	0.27 ± 0.02	0.26 ± 0.07*	39.08 ± 0.01	40.32 ± 0.06*	0.192 ± 0.04	0.189 ± 0.06***
PEG@*siRNA*-PLGA	0.22 ± 0.03	0.23 ± 0.05**	37.11 ± 0.01	37.96 ± 0.02**	0.192 ± 0.02	0.189 ± 0.02***
GalNAc@PEG@*scr-siRNA*-PLGA	0.31 ± 0.01	0.36 ± 0.04	42.08 ± 0.03	42.62 ± 0.05	0.329 ± 0.02	0.390 ± 0.02
GalNAc@PEG@*siRNA*-PLGA	0.23 ± 0.01	0.21 ± 0.03***	36.98 ± 0.03	37.21 ± 0.05**	0.182 ± 0.04	0.190 ± 0.07***

Concentration of creatinine, alanine transaminase and bilirubin in serum of mice of different treatment groups viz., healthy control; untreated control; free siRNA; PEG@*siRNA*-PLGA; GalNAc@PEG@*scr-siRNA*-PLGA; GalNAc@PEG@*siRNA*-PLGA. Data are the means ± SD of three sets of different experiments. Various groups were compared by one-way ANOVA followed by Dunnett’s post hoc test. ***P < 0.001 for NC versus untreated control; *P < 0.05, ***P < 0.001 for free siRNA versus untreated control; **P < 0.01, ***P < 0.001 for PEG@*siRNA*-PLGA versus untreated control. ALT: Alanine transaminase.

#### MTT assay

Different concentrations of GalNAc@PEG@*siRNA*-PLGA were tested for its cytotoxic effect on Huh7 (ASGPR-positive) and MCF7 (ASGPR-negative) cancer cells. As shown in Fig. 4B, GalNAc@PEG@*siRNA*-PLGA inhibited the cancer cell viability in a dose-dependent manner. In comparison with untreated control, 0.1 µM, 0.2 µM and 0.3 µM concentrations of NC exhibited statistically significant cytotoxicity (P < 0.05%, P < 0.01% and P < 0.01%; respectively) towards Huh7 cells. The Huh7 cells were more sensitive towards the NC than MCF7 cells. Tested concentration of 0.2 µM of NC resulted in 50% inhibition of cell viability of Huh7 cells, hence, same concentration was used to determine the effect of free siRNA and other formulations on cancer cells. Free siRNA and PEG@*siRNA*-PLGA also inhibited cell growth to some extent whereas GalNAc@PEG@*scr-siRNA*-PLGA had no effect on viability of the tested cells.

### Biodistribution of GalNAc@PEG@*siRNA*-PLGA

**Figure 5:**
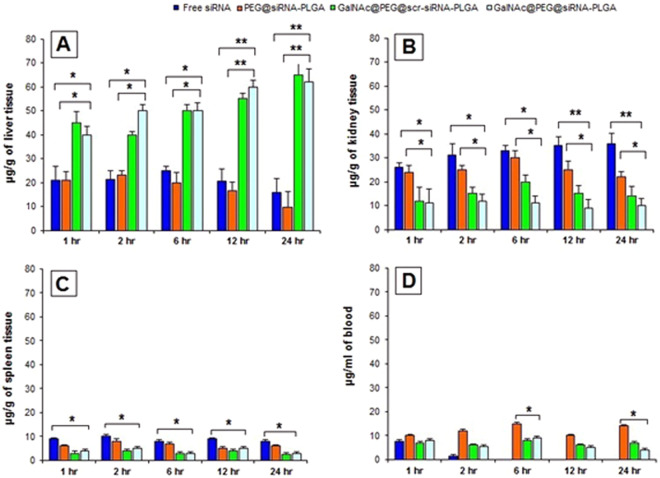
*In vivo* Biodistribution of GalNAc@PEG@*siRNA*-PLGA. The mice were injected intravenously at a single dose of free siRNA, PEG@*siRNA*-PLGA, GalNAc@PEG@scr-*siRNA*-PLGA and GalNAc@PEG@*siRNA*-PLGA. siRNA concentration was determined at 1 h, 2 h, 6 h, 12 h and 24 h time points in Liver **(A)**, Kidney **(B)**, Spleen **(C)** and Blood **(D)**. Data are the means ± SD of three sets of different experiments. Statistical analysis was compared by one-way ANOVA followed by Dunnett’s post hoc test. *P < 0.05, **P < 0.01 for NC versus Free siRNA; *P < 0.05, **P < 0.01 for NC versus PEG@*siRNA*-PLGA.

Survivin siRNA was measured optically for biodistribution of NC in liver, kidney, spleen and blood. As shown in Fig. 5, within one hour of intravenous injection, siRNA carried by NCs was accrued mostly in liver and small portion was carried to kidney. The siRNA concentration of NC was effectively higher in liver in comparison with other organs and blood. Alternatively, the concentration of free siRNA was observed to decrease gradually in liver and increase in kidney demonstrating lesser accumulation of free siRNA in liver and rapid clearance from the body. Likewise, there was decrease in concentration of PEG@*siRNA*-PLGA in liver and increase in kidney. However, after 6 hr, PEG@*siRNA*-PLGA showed decrease in concentrations in kidney. Significantly fewer amounts of siRNA from NC as well as free siRNA accumulated in spleen as compared to liver and kidney. Essentially, at all the time points decrease in siRNA concentration from PEG@*siRNA*-PLGA and NC was observed in blood. Free siRNA was rapidly cleared from blood after 2 hr.

### GalNAc@PEG@*siRNA*-PLGA downregulates survivin mRNA expression

**Figure 6:**
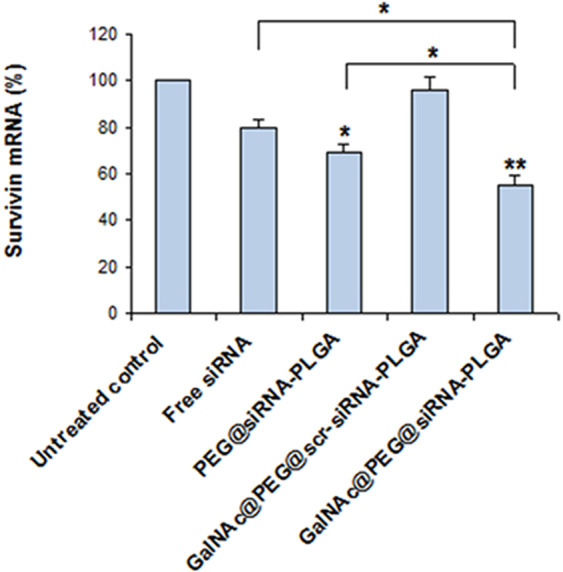
Effect of GalNAc@PEG@*siRNA*-PLGA on mRNA expression. Down-regulation of survivin mRNA levels in liver cancer of mice treated with free siRNA, PEG@*siRNA*-PLGA, GalNAc@PEG@scr-*siRNA*-PLGA and GalNAc@PEG@*siRNA*-PLGA. Data are the means ± SD of three sets of different experiments. Various groups were compared by one-way ANOVA followed by Dunnett’s post hoc test. **P < 0.01 for NC versus untreated control; *P < 0.05 for PEG@*siRNA*-PLGA versus untreated control; *P < 0.05 for NC versus Free siRNA; *P < 0.05 for NC versus PEG@*siRNA*-PLGA.

Figure 6 shows the expression levels of survivin mRNA in treated liver cancer tissues. Survivin mRNA expression was significantly down-regulated in HCC samples of NC treated mice as compared to untreated control (P < 0.01%). in comparison with untreated control, free siRNA (P < 0.05%)and PEG@*siRNA*-PLGA (P < 0.05%) treated tissues showed reduction of survivin mRNA but not to the extent of NC treated tissues. GalNAc@PEG@*scr-siRNA*-PLGA did not cause any change in the mRNA levels and demonstrated results comparable to that of untreated control.

### Histopathological analysis of treated mice

**Figure 7:**
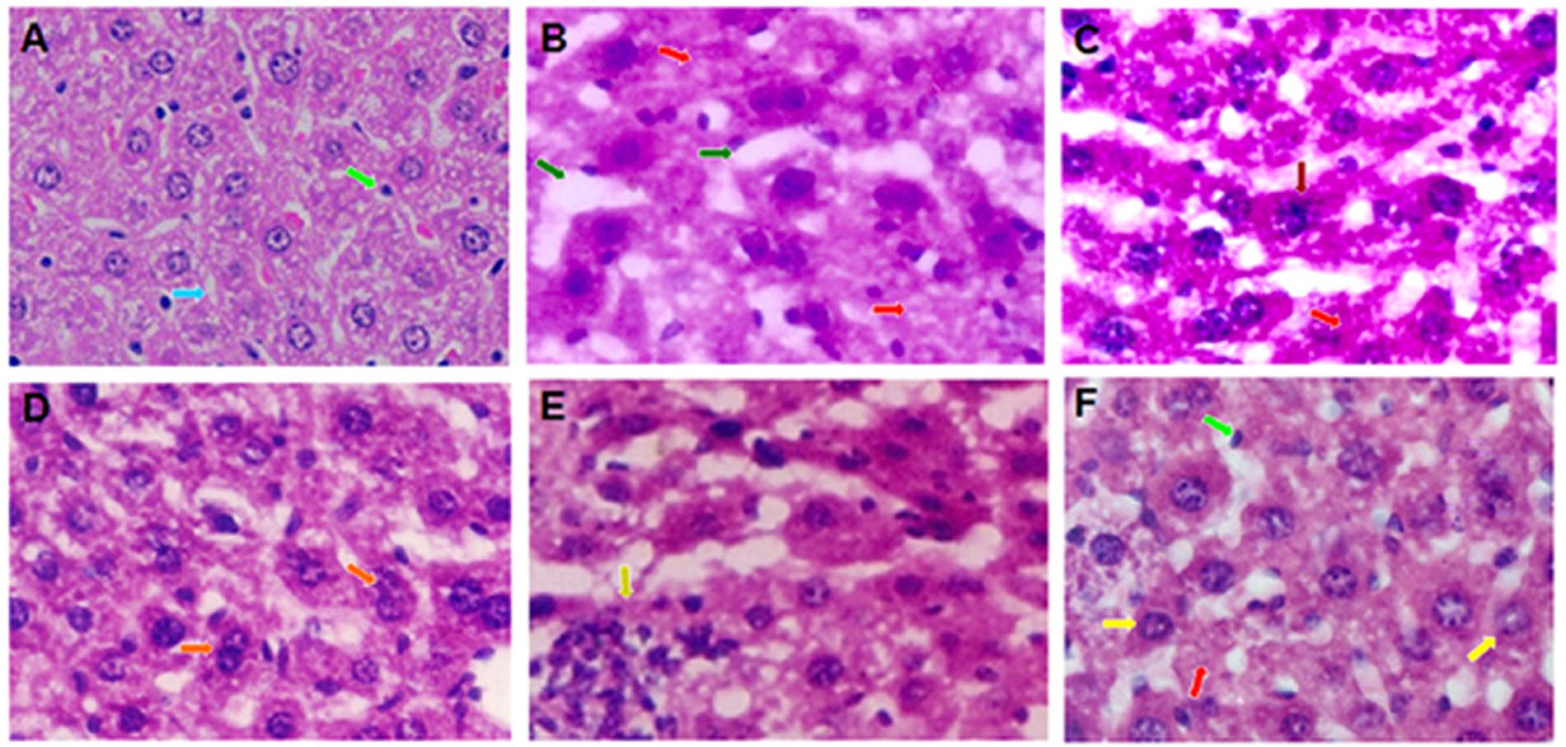
Histopathological studies. Photomicrographs of mouse liver tissue sections from **(A)** healthy control; **(B)** untreated control; **(C)** free siRNA; **(D)** PEG@*siRNA*-PLGA; **(E)** GalNAc@PEG@*scr-siRNA*-PLGA; **(F)** GalNAc@PEG@*siRNA*-PLGA (H&E, 100×). Normal liver structure with sinusoids (), Kupffer cells (), Apoptotic nuclei (), focal lytic necrosis (), enlarged sinusoids (), binuclearity of hepatocytes (), hyperchromatic hepatocytes (), congestion ().

Figure 7 shows the histopathological images of liver cancer tissues of various treated and untreated groups. Liver sections of healthy mice contained normal hepatic laminae, sinusoids and hepatocytes (Fig. 7A). The tissues of untreated control group showed disorganization of normal laminar structure with marked lytic hepatocellular necrosis, distorted sinusoids and laminae (Fig. 7B). Treatment with PEG@*siRNA*-PLGA and free siRNA facilitated to restore the hepatic architecture to a great extent, but still, tumor regression in mice of these groups was lesser than that observed in mice of NC treated group. Treatment with free siRNA showed moderate organization of hepatic laminae with occasional focal lytic necrosis. There was no congestion but obvious hyperchromatic hepatocytes were seen (Fig. 7C). Tissues treated with PEG@*siRNA*-PLGA showed hepatic architecture maintained to a great extent. Occasional focal lytic necrosis and mild increase in the binuclearity of hepatocytes was observed. Moderate increase in kupffer cells were seen (Fig. 7D). The tissues of GalNAc@PEG@*scr-siRNA*-PLGA treated mice showed disorganization of normal hepatic architecture, congestion; infiltration with spared hepatocytes having shrunken and dark nucleus (Fig. 7E). The group treated with NC showed maintained hepatic laminae to a great extent with focal lytic necrosis and apoptosis. No congestion but kupffer cells prominence was seen under the microscope (Fig. 7F).

### Effect of GalNAc@PEG@*siRNA*-PLGA on caspase-3 activation

**Figure 8:**
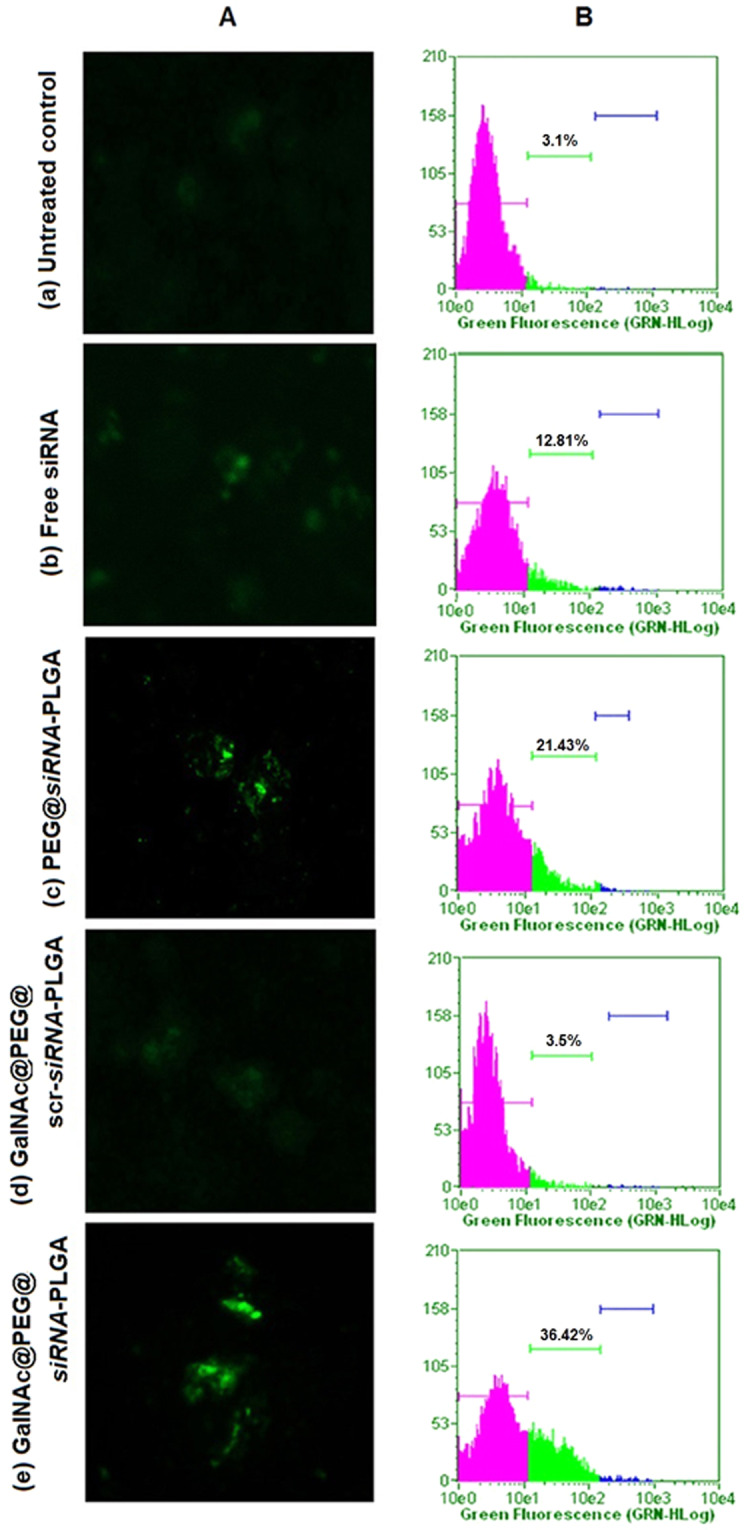
Caspase-3 facilitated apoptosis and DNA fragmentation (**A)** Fluorescence microscopic images depicting involvement of caspase-3 mediated apoptosis in liver cancer cells isolated from various groups viz., (a) untreated control; (b) free siRNA; (c) PEG@*siRNA*-PLGA; (d) GalNAc@PEG@*scr-siRNA*-PLGA; (e) GalNAc@PEG@*siRNA*-PLGA. **(B)** Corresponding graphs shows flow cytometric images of levels of DNA fragmentation in liver cancer cells isolated from various groups as mentioned above. Data are representative of three sets of independent experiments with similar observations.

Figure 8A shows the FITC fluorescent images of single cells from treated liver cancer tissues. In comparison with untreated control (Fig. 8A,a), liver cancer cells of NC treated group showed significantly enhanced expression of caspase-3 (Fig. 8A,e). PEG@*siRNA*-PLGA treated samples also showed significant caspase-3 expression but was not as up-regulated as that of NC treated samples (Fig. 8A,c). The mice treated with free siRNA (Fig. 8A,b) exhibited induction of apoptosis but the expression was lesser than that of the GalNAc@PEG@*siRNA*-PLGA and PEG@*siRNA*-PLGA treated groups. GalNAc@PEG@*scr-siRNA*-PLGA preparation failed to induce caspase-3 activation in liver cancer cells (Fig. 8A,d).

### Effect of GalNAc@PEG@*siRNA*-PLGA on DNA fragmentation

Figure 8B shows flow cytometric analysis of cells undergoing apoptosis in terms of DNA fragmentation. Liver cancer cells of mice treated with GalNAc@PEG@*siRNA*-PLGA revealed significantly increased percentage (36.42%) of DNA fragmentation, as compared to untreated control (3.1%) (P < 0.01) (Fig. 8B,e). Free siRNA (Fig. 8B,b) induced significantly less DNA fragmentation (12.81%) when compared to NC treated mice (P < 0.05%). PEG@*siRNA*-PLGA was able to induce significant fragmentation of DNA (21.43%) but was lesser than NC treated mine (P < 0.05%) (Fig. 8B,c). Additionally, weak DNA fragmentation (3.5%) was observed in GalNAc@PEG@*scr-siRNA*-PLGA treated mice (Fig. 8B,d).

### GalNAc@PEG@*siRNA*-PLGA modulates apoptotic factors

**Figure 9:**
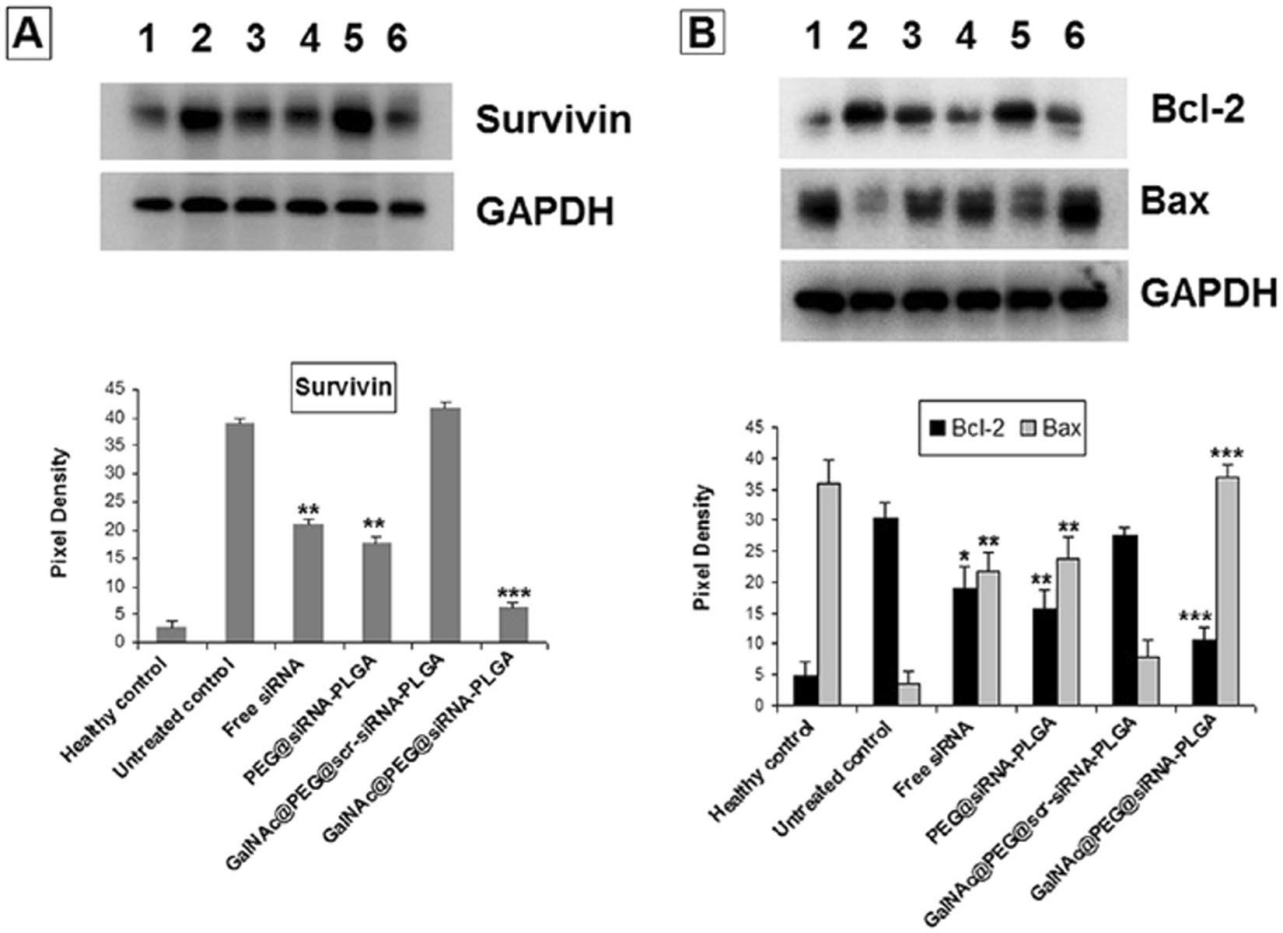
Effect of GalNAc@PEG@*siRNA*-PLGA on Survivin, Bcl-2 and Bax. (**A)** Western blots showing expression of survivin in various treated groups. Lanes: (1) healthy control; (2) untreated control; (3) free siRNA; (4) PEG@*siRNA*-PLGA; (5) GalNAc@PEG@*scr-siRNA*-PLGA; (6) GalNAc@PEG@*siRNA*-PLGA. Data are representative of at least three independent experiments with similar observations. **(B)** Blots showing the effect of NC on the expression of Bcl-2 and Bax proteins. Lanes: (1) healthy control; (2) untreated control; (3) free siRNA; (4) PEG@*siRNA*-PLGA; (5) GalNAc@PEG@*scr-siRNA*-PLGA; (6) GalNAc@PEG@*siRNA*-PLGA. Data are representative of at least three independent experiments with similar observations. Densitograms showing percent expression of Survivin, Bcl-2 and Bax in various groups. Data are the means ± SD of three sets of different experiments. Various groups were compared by one-way ANOVA followed by Dunnett’s post hoc test. ***P < 0.001 for NC versus untreated control; **P < 0.01 for PEG@*siRNA*-PLGA versus untreated control; *P < 0.05, **P < 0.01 for Free siRNA versus untreated control.

First, the tumor samples were analysed for the expression of survivin protein (Fig. 9A). GalNAc@PEG@*siRNA*-PLGA significantly down-regulated the expression of survivin as compared to untreated control. The groups treated with GalNAc@PEG@*scr-siRNA*-PLGA did not exhibit any reduction in survivin levels. However, treatment with free siRNA and PEG@*siRNA*-PLGA caused reduced survivin levels that were quite distinct from untreated control (P < 0.05). The NC revealed highest down-regulation of survivin (P < 0.001) most likely due to the targeted delivery of siRNA to tumor site.

As shown in Fig. 9B, GalNAc@PEG@*siRNA*-PLGA significantly up-regulated the expression of pro-apoptotic protein Bax and down-regulated the expression of anti-apoptotic protein Bcl-2 in comparison with untreated controls (P < 0.001). Treatment with free siRNA and PEG@*siRNA*-PLGA also increased the levels of Bax and decreased the Bcl-2 levels as compared to untreated control but not to a great extent (P < 0.05). Preparation bearing scrambled siRNA did not produce any effect on the expression of pro- or anti-apoptotic proteins.

### Effect of GalNAc@PEG@*siRNA*-PLGA on body weight, weight of vital organs and tumor volume of treated mice

**Figure 10:**
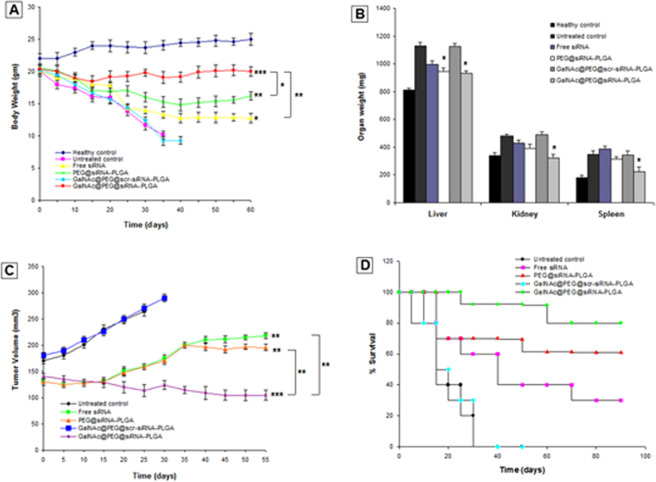
Anti-tumor and survival efficacy in HCC bearing mice of different treatment groups viz., healthy control; untreated control; free siRNA; PEG@*siRNA*-PLGA; GalNAc@PEG@*scr-siRNA*-PLGA; GalNAc@PEG@*siRNA*-PLGA. **(A)** The curves represent total body weight of mice throughout the treatment time period. Data are the means ± SD of three sets of different experiments. Various groups were compared by one-way ANOVA followed by Dunnett’s post hoc test. *P < 0.05, **P < 0.01, ***P < 0.001 for free siRNA, PEG@*siRNA*-PLGA, NC versus untreated control, respectively; **P < 0.01 for NC versus Free siRNA; *P < 0.05 for NC versus PEG@*siRNA*-PLGA; **(B)** The bars represent organ weight of vital organs; liver, kidney and spleen, post-treatment. Data are the means ± SD of three sets of different experiments. Various groups were compared by one-way ANOVA followed by Dunnett’s post hoc test. *P < 0.05 for NC versus untreated control; *P < 0.05 for PEG@*siRNA*-PLGA versus untreated control; **(C)** Growth curves represent the changes in tumor sizes throughout the treatment time period. Data are the means ± SD of three sets of different experiments. Various groups were compared by one-way ANOVA followed by Dunnett’s post hoc test. ***P < 0.001 for NC versus untreated control; **P < 0.01 for free siRNA and PEG@*siRNA*-PLGA versus untreated control; **P < 0.01 for NC versus Free siRNA and PEG@*siRNA*-PLGA; **(D)** Kaplan-Meier curve showing the efficacy of GalNAc@PEG@*siRNA*-PLGA in terms of survival at different time points post-treatment. Data are the means ± SD of three sets of different experiments.

Body weights were also measured on a weekly basis during the course of survival studies (Fig. 10A). The treatment with GalNAc@PEG@*siRNA*-PLGA prevented significant weight loss and showed restoration of weight to almost 90% of body weight when compared with untreated control (P < 0.01). Free siRNA and PEG@*siRNA*-PLGA treated mice alleviated the weight loss to (60%; P < 0.05) and (75%; P < 0.05), respectively; but not as comparable to that of NC. GalNAc@PEG@*scr-siRNA*-PLGA treated mice could not restore their body weight and showed almost 30% reduction in body weight that resulted in mortality by fifth week, post-treatment. Liver, kidney and spleen were swollen and enlarged in experimental HCC mice. NC treatment resulted in significantly good recovery and improvement of organomegaly of vital organs (Fig. 10B). Treatment with free siRNA and PEG@*siRNA*-PLGA also improved the organ mass but was not able to induce protection as compared with NC. GalNAc@PEG@*scr-siRNA*-PLGA treated mice did not show similar recovery of organ mass. At the end of the resting phase, as shown in Fig. 10C, mice of untreated group had mean tumor volume of 265 ± 15 mm^3^. Treatment with NC significantly reduced this growth to 105 ± 08 mm^3^ (P < 0.001). PEG@*siRNA*-PLGA significantly reduced the tumor volume to 196 ± 10 mm^3^ (P < 0.01) than that for the untreated and the free siRNA (218 ± 14 mm^3^) groups (P < 0.01), possibly due to passive targeting of tumor tissues. Notably, among these formulation groups, GalNAc@PEG@*scr-siRNA*-PLGA had no effect at all.

### Effect of GalNAc@PEG@*siRNA*-PLGA on survival rate of treated mice

The anticancer efficacy of different tested formulations on experimental HCC at different time points is shown in Fig. 10D. Survival was monitored for 12 weeks following the beginning of treatment. The mice treated with GalNAc@PEG@*siRNA*-PLGA during 12 weeks duration demonstrated 80% survival (P < 0.001). Treatment with free siRNA and PEG@*siRNA*-PLGA resulted in 30% and 60% survival (P < 0.05), respectively; while those administered with GalNAc@PEG@*scr-siRNA*-PLGA did not survive beyond 5 weeks. Similarly, liver cancer bearing mice of untreated group succumbed to death by fifth week post-treatment.

## Discussion

Among various cancer related proteins, survivin has been identified as one of the most specific proteins. It is a member of apoptosis inhibitor protein family that plays a pivotal role in cancer progression and proliferation by inhibiting apoptosis^33–35^. Since it is highly overexpressed in majority of human cancers, including HCC and is undetectable in normal tissues, its inhibition has been tracked as a convincing strategy for cancer therapy^33,36,37^. Survivin targeted siRNA has shown effectiveness in inducing apoptosis in tumor cells^38^. Both *in vitro* and *in vivo s*tudies have shown the inhibition of survivin expression by RNAi therapy resulting in growth inhibition of HCC cells^39^. Hepatocytes are predominantly important target cells towards siRNA delivery because of their ability to be accessed directly by the nanoparticles even after simple intravenous injection. However, due to passive targeting of siRNA to the liver^40^, they are delivered to other non-target cells resulting in toxicity. To avoid non-specific interactions, the delivery vehicles can be attached to hydrophilic non-interactive agents like PEG^41^. Moreover, to specifically target hepatocytes and enhance the cellular uptake of the nanoparticles various ligands can be conjugated for targeting overexpressed cell surface receptors present on the HCC.

Here, we demonstrate a successful development of siRNA encapsulated multi-functional NC for selective knockdown of gene of interest in HCC cells. The synergistic antitumor efficacy of engineered GalNAc functionalized survivin siRNA loaded PEGylated PLGA (GalNAc@PEG@*siRNA*-PLGA) NCs was evaluated in mouse HCC. The stability of the NC with intact siRNA is necessary for accurate crossing over the cellular barriers and successful delivery of siRNA at the target site for potent silencing of the specific gene. Characterization of GalNAc@PEG@*siRNA*-PLGA employing SEM, TEM, zeta potential, PDI and entrapment efficiency confirmed the formation of stable NC. According to SEM results, siRNA encapsulated PLGA nanoparticles were of an average size of 190 ± 20 nm. A slight increase in an average size from 190 nm to 210 nm (TEM) confirmed the successful anchoring of GalNAc ligands on the surface of PEG coated PLGA nanoparticles (Fig. 1B,C). Structurally, in-house designed NCs were compact and spherical with a rough surface. The uneven surface was possibly due to the presence of ligand on the PEGylated PLGAs. They had favourable zeta potential of −6.7 ± 0.4 mv, PDI of 0.108, entrapment of about 53 ± 0.5% and loading efficiency of 690 ± 28 ng/mg NC. The NCs also demonstrated a biphasic pattern of siRNA release with an initial burst release followed by a slow and sustained release (Fig. 1D). In the present study, about 25% of siRNA was released as a result of burst release. The initial burst release denotes release of siRNA that was associated on the surface of a polymer. This also indicates that rest of the 75% is localized inside the matrix of the NC. The change in the release profile of remaining siRNA can be ascribed to the change in matrix degradation rate^25^. Sustained and slow release of NC for a period of 15 days could be ascribed to the polymeric matrix that provides a barrier to siRNA release and preserves siRNA for prolonged time period.

The targeting specificity of PEG@*siRNA*-PLGA decorated with ASGPR specific GalNAc ligand was established on HCC cells. Specificity was evaluated on Huh7 (ASGPR-positive) and MCF7 (ASGPR-negative) cell lines. MCF7 cells were taken because of their two prominent features. First, MCF7 breast cancer cells possess high endocytic activity and second, they are reported to be used as control in studies where selectivity of galactosylated carriers is tested towards ASGPR^42^. Punctate fluorescence localized on the periphery of the ASGPR-positive Huh7 cells suggested accumulation of NCs on the surface of liver cancer cells (Fig. 3A). Absence of fluorescence on MCF7 (ASGPR-negative) cells approves the specific targeting by GalNAc@PEG@*siRNA*-PLGA. Incubation of these cells with PEG@*siRNA*-PLGA formulation also supports the specific role of GalNAc as an important targeting agent. These results also suggest the significance of GalNAc ligation that played an important role in specifically inhibiting viability of ASGPR-positive Huh7 cells. Moreover, competitive inhibition of the uptake of NC specifies that GalNAc decorated PEG@*siRNA*-PLGA nanoparticles were able to successfully target the ASGPR-expressing HCC cells (Fig. 3B). This also suggests that other endocytic pathways are not involved in the uptake of GalNAc@PEG@*siRNA*-PLGA NCs.

The results also determined the *in vitro* and *in vivo* treatment associated toxicity of the NCs. The toxicity studies in healthy mice indicated no toxicity in hepatic and renal systems associated with NC administration (Table 1). The NCs were also non-toxic to RBCs (Fig. 4A). When NC was tested for its cytotoxicity against ASGPR-positive and ASGPR-negative cancer cell lines, the effect was distinct. The treatment with GalNAc@PEG@*siRNA*-PLGA resulted in significant cytotoxicity in Huh7 (ASGPR-positive) cells whereas no effect was observed on MCF7 (ASGPR-negative) cells (Fig. 4B). These results also suggest the significance of GalNAc ligation that played an important role in specifically inhibiting viability of ASGPR-positive Huh7 cells. *In vivo* propagation is usually been determined by examining the biodistribution of the payload entrapped within the nanoparticles^43^. siRNA content was found to be significantly higher in liver of mice administered with NC than its free form (Fig. 5). This high accumulation of siRNA in liver and reduced distribution in other organs can be ascribed to specific targeting by GalNAc towards liver cancer cells.

The efficacy of encapsulated survivin siRNA to knockdown the target gene survivin upon delivery within the hepatocytes of HCC bearing mice was validated by the expression of survivin mRNA. There was significant down-regulation of survivin mRNA thereby confirming the transfection of HCC cells by NCs (Fig. 6). Knockdown of survivin mRNA was also backed by considerable decrease in expression of survivin protein by approximately 60% levels (Fig. 7A). The results clearly demonstrated that transfection of HCC cells with survivin siRNA encapsulated NC significantly down-regulated the mRNA and, as vizualized using RT-qPCR and Western blot analysis (Figs 6 and 8A, respectively). These results indicate effective silencing of the targeted survivin gene by GalNAc@PEG@*siRNA*-PLGA into the tumor cells.

The survivin gene plays an important role in regulating apoptosis. It has been shown that survivin directly binds to caspase-3 and caspase-7 inhibiting their activities thereby resulting in suppression of apoptosis^44^. Knockdown of survivin gene using anti-sense oligonucleotides, genetic deletion or siRNAs have resulted in induction of apoptosis in tumor cells^45–47^. In the present study, using NC as a therapeutic agent, we were able to successfully knock down elevated survivin siRNA at mRNA as well as protein level in HCC cells (Figs 6 and 8A). It was observed that survivin mRNA and protein expression was significantly decreased upon treatment. Free siRNA and PEG@*siRNA*-PLGA were also able to decrease the expression of elevated survivin levels but were not comparable to GalNAc@PEG@*siRNA*-PLGA treated group. In GalNAc@PEG@scr-siRNA-PLGA system, because of the presence of GalNAc, the NC encapsulating scrambled siRNA was able to reach and incorporate into the HCC cells of treated mice but was ineffective and did not reduced the level of survivin protein. Since, survivin inhibition has been associated with apoptosis induction and down-regulation of survivin mRNA and protein levels by in-house synthesized GalNAc@PEG@*siRNA*-PLGA, we expanded our study to determine the effect of NCs on the induction of apoptosis. Definite evidence of apoptosis induction by the NC was assessed by analysing caspase-3 expression, induction of DNA fragmentation and expression of pro- and anti-apoptotic molecules. Increase in caspase-3 fluorescence and increase in green signal signifying DNA breaks collectively suggested the apoptotic induction in NC treated HCC cells (Fig. 8A,B). Bcl-2 is one of the important apoptotic proteins that play a role in progression of cancer. It is reported to be an integral part of malignancies and is a vital component in directing cell death^48^. In HCC cells, Bcl-2 expression is raised that initiates cancer pathogenesis^49^. Apoptotic induction in cancer cells upon treatment by chemotherapeutic agents is primarily governed by Bcl-2-Bax ratio^50^. In the present study, GalNAc@PEG@*siRNA*-PLGA significantly induced and modulated cellular apoptosis as confirmed by regulation of Bax and Bcl-2 expression. Free siRNA also modulated the expression of tested pro- and anti-apoptotic proteins but the magnitude was mediocre to the effect of siRNA encapsulated within the delivery system (Fig. 9B). PEG@*siRNA*-PLGA also significantly induced apoptosis as evident by various tested parameters but was lesser than the specifically targeted NCs. It is worth mentioning that the targeted delivery of the NCs to the ASGPR receptors of HCC cells vividly heightened its anticancer ability.

The higher efficacy of GalNAc@PEG@*siRNA*-PLGA was also verified by histopathological alterations, changes in whole body weight, weight of vital organs and survival rate of treated mice. The NC treated cancerous hepatocytes demonstrated profound recovery and were able to restore the cellular architecture to normal form (Fig. 7). Moreover, administration of the NC caused significant alleviation of the loss of whole body weight as well as those of vital organs (Fig. 9A,B). The mice treated with GalNAc@PEG@*siRNA*-PLGA were able to survive for a period of 12 weeks as compared to those treated with free siRNA (Fig. 10D).

## Conclusion

In the present study, the linking of targeting ligand significantly improved the therapeutic index of the molecular therapy in HCC bearing mice. Our data shows that GalNAc@PEG@*siRNA*-PLGA was able to efficiently bind to ASGPR overexpressing at HCC cells and increased the uptake of survivin siRNA. GalNAc@PEG@*siRNA*-PLGA NCs validated superior anti-tumor effect with respect to free survivin siRNA which may be because of the following parameter viz., galactosylation of GalNAc to PLGA enhanced the cellular uptake and internalization of NC into hepatoma cells; PEGylation increased the accumulation of siRNA in tissue by prolonging the circulation time and reducing uptake by reticuloendothelial system. Moreover, the nanosize of the NC facilitated easier infiltration of HCC by means of enhanced permeation.

## Materials and Methods

### Chemicals

Survivin specific siRNA was supplied by Santa Cruz Biotechnology, Inc. (CA, USA). PolyDL-lactic-co-glycolic acids (50:50) (PLGA), N-acetylgalactosamine (GalNAc), fluorescein isothiocyanate (FITC), diethylnitrosomanine (DEN), trizol reagent and Dulbecco’s Minimal Essential Medium (DMEM), Asialofetuin (AF) were purchased from Sigma-Aldrich Inc. (St. Louis, MO). Alexa Fluor 488 was obtained from Molecular Probes. Polyethylene-maleic anhydride (PEMA) and Fmoc-PEG-COOH were bought from Polysciences, Inc. and Nektar (San Carlos, CA), respectively. Polyvenylidene difluoride (PVDF) membranes and sterile filters (0.22 µm) were obtained from Millipore (Germany). All the chemicals used in this study were of highest purity.

Antibodies viz., anti-Bcl-2, anti-Bax, anti-caspase-3, anti-survivin, GAPDH and BrdU TUNEL assay kit were purchased from BD Biosciences (San Diego, CA). Goat anti-mouse IgG–horseradish peroxidase secondary antibody was purchased from Amersham Pharmacia Biotech (Uppsala, Sweden). Rabbit anti-mouse FITC tagged secondary antibody was purchased from Sigma-Aldrich Inc. (St. Louis, MO).

Huh7, human hepatocellular carcinoma cell line was procured from Thermo Fisher Scientific and MCF7, human breast adenocarcinoma cell line was purchased from ATCC, (Rockville, MD Inst.).

### Mice

Swiss albino mice (Male; 22 ± 2 g body weight) were acquired from animal house facility of King Saud University, Riyadh. Mice were quarantined and acclimated for a week under standard atmospheric conditions of 22 ± 1 °C temperature and 50–60% humidity. Mice were given food and water as per requirement. The study was done in accordance with the principles of National Institute of Health Guide for the Care and Use of Laboratory Animals

(NIH Publications 8^th^ edition; 2011). The study was approved by the animal facilities guidelines from the Ethical committee of Experimental Animal Care Center, College of Pharmacy, King Saud University (Clearance No. 5695; January, 2016).

### Induction of HCC in mice

Mice were injected with 2.4 mg/mouse of diethylnitrosamine (DEN) intraperitoneally, for the induction of HCC^24^. During induction time mice were fed normal food. After a period of 40 days, formation of HCC was confirmed histopathologically and by estimation of liver enzymes.

### Preparation of siRNA loaded PLGA nanoparticles

The siRNA loaded PLGA nanoparticles were prepared by the double emulsion (water-in-oil-in-water) solvent evaporation method of Cun *et al*.^25^ with some modifications. Firstly, survivin siRNA (1 mg) was mixed in 0.5 ml of dichloromethane and acetone (3:1 v/v) solution containing 100 mg of PLGA. This mixture was dissolved in 0.5 ml of 1.5% polyvinyl alcohol (PVA) solution and emulsified into a primary w/o emulsion by sonication for 30 s. To this, 4 ml of 1.5% PVA was added and the primary emulsion was further sonicated for 60 s to form a w/o/w double emulsion. The subsequent double emulsion comprising of *siRNA*-PLGA nanoparticles was diluted with 20 ml of 0.15% PVA and then magnetically stirred for 6 h at RT to evaporate the solvent.

For PEGylation, after the preparation of *siRNA*-PLGA nanoparticles, the carboxylic acid present on PLGA was functionalized with PEG. To increase the carboxyl groups on the surface of PLGA, Polyethylene-maleic anhydride (PEMA) was used as a surfactant. Briefly, *siRNA*-PLGA nanoparticles (50 mg) were firstly dissolved in acetone (3 ml) and then were mixed into PEMA (0.2%) under stirring (200 rpm). Modified *siRNA*-PLGA nanoparticles were then collected by centrifugation (15,000 g for 10 min) and washed three times with double distilled water.

### Preparation of PEGylated-*siRNA*-PLGA nanoparticles

Pegylation was done according to the protocol of Zhang *et al*.^26^. Briefly, Fmoc-PEG was stirred in 1.5 ml 20% piperidine in dimethylformamide for 2 h at RT to de-protect the amine side of PEG. Water was added to the solution and solution was then centrifuged and filtered for complete removal of Fmoc. The solution was extensively dialysed again and then lyophilized. For the synthesis of PEGylated PLGA, 5 mg of PEMA treated *siRNA*-PLGA nanoparticles (~0.2 mg/ml double-distilled water) were incubated with 23 mg of 0.2 mmol N-hydroxysuccinimide (NHS, pH 5.8). To it, 153 mg of 0.8 mmol 1-(3-dimethylaminopropyl)-3-ethylcarbodimide hydrochloride (EDC) was added and the mixture was incubated with gentle stirring for 2 h at RT. The resulting NHS-activated nanoparticles were covalently linked to 10 mg NH-PEG-COOH. The resulting *siRNA*-PLGA-PEG-COOH nanoparticles were again washed, resuspended, and preserved in suspension form in double-distilled water.

### Conjugation of N-acetylgalactosamine to *siRNA*-PLGA-PEG-COOH nanoparticles

*N*-acetylgalactosamine (GalNAc) was conjugated to PEGylated nanoparticle according to the published protocol^26^. The *siRNA*-PLGA-PEG-COOH nanoparticles were activated using NHS as mentioned above. The activated *siRNA*-PLGA-PEG-COOH nanoparticles were covalently linked to GalNAc. PEG-decorated PLGA nanoparticles were washed twice in 1 M NaCl to remove physically adsorbed polymer and then with double distilled water. The resulting GalNAc@PEG@*siRNA*-PLGA nanoconjugates (NCs) were subsequently lyophilized and stored at −20 °C until further use.

### Quantification of GalNAc on the surface of PEG@*siRNA*-PLGA

The unreacted PEG and GalNAc were separated from nanoparticles after centrifugation at 15,000 rpm for 45 min. The amount of free PEG and GalNAc in the supernatant was determined using 2,4,6-trinitrobenzenesulphonic acid (TNBS) as a colorimetric assay according to the published protocol^27^. The absorption was determined at 420 nm on a UV/VIS spectrofluorometer (Jasco, FP-8200). The amount of PEG and GalNAc conjugated on the surface of *siRNA*-PLGA and PEG@*siRNA*-PLGA respectively, was calculated by subtracting the free amount from the total amount added into the reaction system.

### Characterization of GalNAc@PEG@*siRNA*-PLGA

#### Surface Electron Microscopy

The size and surface morphology of NCs was determined by scanning electron microscopy (SEM). Briefly, lyophilized NCs were re-suspended in PBS, pH 7.4. A drop of NC suspension was mounted on clean gold palladium alloy coated glass stub and imaged on SEM (Zeiss EVO 40; Carl Zeiss SMT AG, Oberkochen, Germany).

#### Transmission Electron Microscopy

A drop of NCs was mounted over gold coated negative grid of transmission electron microscope (Model HT 7700, Hitachi High Technologies, America Inc.) followed by evaporation of the solvent. The analysis was performed at an accelerating voltage of 200 kV.

#### Polydispersity index and zeta-potential

Freeze-dried NCs (0.2 mg/ml) were dispersed in double distilled water by short gentle sonication. The average polydispersity index (PDI) and zeta-potential of GalNAc@PEG@*siRNA*-PLGA NCs were determined using a dynamic light scattering (DLS) detector, Zeta Nano ZS (Malvern Instruments Ltd., Worcestershire, UK). Cumulant analysis method was adopted for analysing the PDI values whereas zeta-potential was measured according to Smoluchowski equation. The measurements were performed in triplicates on undiluted samples at 25 °C.

#### Entrapment efficiency and loading amount of siRNA in GalNAc@PEG@siRNA-PLGA

Firstly, freeze-dried NCs (2 mg) were dissolved in 200 µl of chloroform per sample. For extraction of siRNA from organic phase to aqueous phase, TE buffer (500 µl) was added and the mixture was gently rotated for 90 min at RT. Now, separation of both the phases was done by centrifugation of mixture at 15,000 g at 4 °C for 20 min. The supernatant (aqueous phase) samples were then diluted more with TE buffer. The siRNA concentration was determined using NanoDrop at a wavelength of 260 nm. The entrapment efficiency and loading amount were calculated according to the equation provided by Cun *et al*.^28^.

#### Release of siRNA from GalNAc@PEG@siRNA-PLGA

The release of siRNA was measured according to the protocol of Cun *et al*.^28^. Briefly, 2.0 mg of freeze-dried GalNAc@PEG@*siRNA*-PLGA was suspended in 1 ml of PBS (pH 7.4) buffer in RNase-free Eppendorfs. The samples were gently stirred in a water bath at 37 °C. According to the protocol the aliquots at different time intervals were saved, centrifuged and the supernatants were collected for analysis. The release kinetics was continued for 15 days and the samples were analysed in triplicates.

#### Targeting efficiency of GalNAc@PEG@siRNA-PLGA

Cell specific targeting of the NCs was established according to the published protocol^26^. Initially, GalNAc@PEG@*siRNA*-PLGA was activated by NHS as mentioned under the sub-heading “Conjugation of *N*-acetylgalactosamine to *siRNA*-PLGA-PEG-COOH nanoparticles”. Now, NHS-activated NCs were covalently linked to 0.2 mg Alexa-488. Freshly prepared fluorescently-labeled NCs were diluted with serum free medium at a concentration of 0.5 mg/ml. A human hepatocellular carcinoma cell line, Huh7 (ASGPR-positive) and a human breast adenocarcinoma cell line, MCF7 (ASGPR-negative) were used to monitor the specific binding of GalNAc@PEG@*siRNA*-PLGA to ASGPR receptors^21^. Both types of cells at a concentration of 1 × 10^5^ each were seeded in 24-well plates in DMEM medium supplemented with penicillin (100U), streptomycin (100 mg) and FBS (10%) at 37 °C under 5% CO_2_ for 24 h. For surface binding, the fluorescently-labeled NCs were added to the cells and incubated at 37 °C for 2 h. The cells were washed thrice with PBS and NC content was determined by visualizing the Alexa-488 fluorescence using a fluorescence microscope (EVOS, Life Technologies, 100X magnifications). Both of the cell lines were also incubated with fluorescently-labeled PEG@siRNA-PLGA formulation accordingly as mentioned above.

To confirm the ASGPR-mediated cell binding of NC in Huh7 cells, asialofetuin (AF) or GalNAc was used as an inhibitor in separate sets of competitive experiments. NCs were fluorescently-labeled and Huh7 cells at a concentration of 5 × 10^5^ were seeded as mentioned above. For studies, Huh7 cells were pre-incubated with increasing concentrations of AF (0–10 µM) or GalNAc (0–100 mM) for 30 min at 37 °C, washed thrice and then incubated with Alexa-488 labeled NCs. After 30 min treatment of NCs, cells were washed and analysed. Fluorescence in each wells were determined at 620 nm in Elisa plate reader.

#### Toxicity tests for GalNAc@PEG@siRNA-PLGA

Hemolysis testing: Initial acute toxicity of NCs was tested through *in vitro* erythrocyte lysis test^29^. Here, the hemoglobin, released as a result of membrane leakage or disruption caused by exposure to high doses of the drug was measured spectrophotometrically. Fresh blood from a healthy rabbit was collected in anticoagulant solution and subjected to centrifugation at 1,000 g for 15 min at 4 °C. Buffy coat as well as plasma was discarded. The washed erythrocytes were diluted with isotonic buffer (10 mM phosphate buffer, 150 mM NaCl) and 50% hematocrit was prepared. To study the extent of haemolysis, the suspension of RBCs was incubated with 100 µg/ml of various siRNA loaded NC formulations or free siRNA at 37 °C for 1 h. After 1 h, the reaction mixture was centrifuged at 1,500 g and the supernatant was collected and analyzed by UV-Visible spectroscopy (λmax = 576 nm) for released hemoglobin.

*In vivo* toxicity: Renal and hepatic toxicities were evaluated by applying multi-dose regimen with various siRNA loaded NC formulations or free siRNA^30^. A total of three dose regimens (single dose of 300 µg/healthy mouse at days 1, 3 and 5) were applied and toxicity levels in the liver and kidney of administered mice were monitored by determining the concentrations of serum creatinine, serum alanine transaminase and total bilirubin. At days 0 (pre-dose) and 6 (post-dose) of intravenous administration, the blood was taken from the retro-orbital region of mice from different groups. The serum separated from clotted blood was used for determining creatinine, alanine transaminase and bilirubin levels according to the manufacturer’s protocol. Healthy mice were taken as positive control whereas liver cancer bearing mice were taken as untreated negative control.

MTT assay: Cytotoxicity on liver cancer cell lines; Huh7 (ASGPR-positive) and MCF7 (ASGPR-negative) was examined by performing MTT assay. Briefly, cells at a density of 1 × 10^5^/well were seeded in 96-well plates and grown in their respective medium in the presence of 5% FCS for 24 h at 37 °C. The cells were then separately treated with 0.05 µM, 0.1 µM, 0.2 µM and 0.3 µM concentration of NC for 94 h using the same culture conditions. After incubation period, cell proliferation was measured by adding 5 mg (per ml PBS) of MTT dye in each well. The plates were incubated for 4 h at 37 °C in a humidified chamber containing 5% CO_2_. Formation of Formazan crystals in the reaction mixture was observed by dissolving them in 100 µl of DMSO. Absorbance was read at 620 nm in multi-plate reader and absorbance values were expressed in terms of cell viability (%) with reference to the untreated control group taken as 100%. For comparison, Huh7 and MCF7 cell lines were also tested for cytotoxicity levels generated by free siRNA and other siRNA bearing formulations at a concentration of 0.2 µM.

### *In vivo* biodistribution of GalNAc@PEG@*siRNA*-PLGA

Biodistribution studies were performed according to Sherwani *et al*.^31^. Free siRNA and various siRNA loaded NC formulations were injected separately in mice, intravenously. *In vivo* distribution was recorded at 1 h, 2 h, 6 h, 12 h and 24 h time points post-injection. For the presence of siRNA in systemic circulation, blood was collected by retro-orbital puncture at selected time periods. Blood was centrifuged at 1000 × g for 10 minutes. Collected serum was precipitated and further centrifuged at 10,000 × g for 10 min. Supernatant was examined for siRNA content by using NanoDrop at a wavelength of 260 nm. Various vital organs like liver, kidney and spleen were also isolated for studying the biodistribution of NC. Mice were sacrificed at selected time periods, and vital organs were removed aseptically. Organs were washed, dried, weighed and homogenized. Tissue lysate was centrifuged at 10,000 × g and supernatant was collected. siRNA content was analysed from supernatant using NanoDrop at a wavelength of 260 nm.

### Treatment regimen of HCC

After induction of liver cancer, mice with an average body weight of 20 ± 0.1 g were randomized into five groups (20 mice/group). Group I was taken as untreated control where HCC bearing mice received no treatment. HCC bearing mice of Group II, III, and IV received treatment in the form of injection of free siRNA, siRNA loaded PEGylated PLGA (PEG@*siRNA*-PLGA) and ligand decorated scrambled siRNA loaded PEGylated PLGA (GalNAc@PEG@*scr-siRNA*-PLGA), respectively. Group V mice were administered with ligand decorated siRNA loaded PEGylated PLGA (GalNAc@PEG@*siRNA*-PLGA) NCs. Each injection comprising of 150 nM survivin (or scrambled) siRNA/200 µl of PBS/animal was administered intravenously once in a day for 10 days consecutively. The therapeutic effect of NC was determined on changes in body weight, weight of organs, change in tumor volumes and survival capacity during 12 weeks post-treatment. Average tumor volumes from each group were measured using vernier caliper after sacrificing the mice.

### *RT-qPCR* for analysis of survivin mRNA expression

Total RNA from cancerous liver tissue of treated mice was isolated using Trizol reagent according to the manufacturer’s protocol. cDNA synthesis and subsequent polymerization was performed using a SensiMix™ SYBR One-Step kit (Quantace; Bioline Reagents Ltd., London, UK). Reverse transcription-quantitative polymerase chain reaction (RT-qPCR) was performed on the ABI PRISM real-time PCR system (Applied Biosystems; Thermo Fisher Scientific, Inc.) using 2xOne-Step qPCR Mix (12.5 µl), 50x SYBR Green I (0.5 µl), primers (0.5 µl), and cDNA template (100 ng, 4 µl). Primers used are:

Survivin-forward sequence: 5′-ACGACCCCATAGAGGAACAT-3′

Survivin-reverse sequence: 5′-TCCGCAGTTTCCTCAAATTC-3′,

GAPDH-forward sequence: 5′-GAAGGTGAAGGTCGGAGTC-3′,

GAPDH-reverse sequence: 5′-GAAGATGGTGATGGGATTTC-3′.

The thermocycler conditions were as follows: Pre-incubation at 95 °C for 10 min, followed by 45 amplification cycles at 95 °C for 20 sec, 58 °C for 30 sec, and 72 °C for 30 sec. The housekeeping gene GAPDH was used as an internal control. Relative gene expression was calculated by the 2ΔΔCq method^32^.

### Histopathological studies

The treated cancerous liver tissue samples were processed according to the protocol of Khan *et al*.^30^. Histopathological slides were observed under a light microscope (Olympus CLX 41) at 100X magnification.

### Fluorescence microscopy for examining caspase-3 expression

The preparation of single cell suspension from treated cancerous liver tissue samples was done according to the published protocol^6^. Briefly, single cell suspension after treatment with 0.1% Triton X-100 in PBS for 10 min was fixed in 4% paraformaldehyde for 2 h at room temperature. The fixed cells were blocked with 2% fetal calf serum, incubated with caspase-3 monoclonal antibody followed by incubation with FITC tagged secondary antibody. Presence of caspase-3 was detected using fluorescence microscope (EVOS, Life Technologies, 100X magnifications).

### DNA fragmentation analysed by TUNEL assay

Single-cell suspensions from treated cancerous liver tissue samples were developed as per the published protocol^6^. Briefly, liver tumor cells were fixed in 1% paraformaldehyde in PBS (7.4 pH) after centrifugation at 300 g for 10 min at 4 °C. Fixed cell suspension was kept on ice for 30 min followed by centrifugation at 300 g for 5 min. The cell pellet was washed with PBS twice and cell concentration was adjusted to 1–2 × 10^6^ cells/ml in 70% ice cold ethanol. After 30 min, cells were washed and resuspended in 50 ml of DNA labelling solution (Reaction buffer + TdT enzyme and BrdUTP). Cells were incubated in the DNA labelling solution for 60 min at 37 °C. After incubation, cells were rinsed, centrifuged and incubated with fluorescein isothiocyanate-conjugated anti-bromodeoxyuridine (FITC-BrdU) antibody in dark for 30 min. After labelling, 50 µg/ml PI/RNase was added to antibody-labeled cells and incubated at 37 °C for 30 min further. Finally, the labeled tumor cells were analyzed by a flow cytometer (MACSQuant, Germany).

### Western blot analysis

The liver tissue samples from treated mice were homogenized in the presence of a protease inhibitor cocktail. A nuclear fraction was extracted according to the procedure of Khan *et al*.^30^. Proteins in the nuclear fraction was estimated by NanoDrop 2000 (Thermo Scientific, USA) using a bovine serum albumin as protein standard. For Western blotting, 30 µg of protein samples was resolved on 10% SDS-PAGE and then electroblotted onto PVDF membranes. The membranes were immunologically probed for the presence of survivin, Bax and Bcl-2 proteins using specific antibodies. Immunoblots were further incubated in horseradish peroxidase-conjugated anti-mouse IgG secondary antibodies for 1 h and immuno-reactive bands were visualized employing enhanced chemiluminescence detection kit (Bio Rad Laboratories, Inc., CA, USA). The membranes were re-probed with GAPDH antibody for enumerating equal protein loading. The expression frequency of proteins was measured by density of bands.

### Statistical analysis

Results are expressed as mean ± SD. Data were analysed and two groups were compared with the Student t-test. Multiple groups at the same time points were compared using ANOVA followed by Dunnett’s post-hoc test. A P values of <0.001, <0.01 and <0.05 were considered to denote statistically significant difference. The statistical analysis was done using Sigma-Plot 10 v software.

## Supplementary information

Supplementary Information.
