# Author Correction: Therapeutic potential of functionalized siRNA nanoparticles on regression of liver cancer in experimental mice

**DOI:** 10.1038/s41598-022-25307-x

**Published:** 2022-12-09

**Authors:** Azmat Ali Khan, Amer M. Alanazi, Mumtaz Jabeen, Arun Chauhan, Mohammad Azam Ansari

**Affiliations:** 1 Pharmaceutical Biotechnology Laboratory, Department of Pharmaceutical Chemistry, College of Pharmacy, King Saud University, Riyadh, 11451 Saudi Arabia; 2 Aligarh, India; 3 Department of Neuroimmunology, School of Health and Medicine, University of North Dakota, Grand Forks, ND USA; 4 Department of Epidemic Disease Research, Institutes of Research and Medical Consultations (IRMC), Imam Abdulrahman Bin Faisal University, 31441 Dammam, Saudi Arabia

Correction to: *Scientific Reports* 10.1038/s41598-019-52142-4, published online 01 November 2019

The original version of this Article contained errors.

The full blots were omitted and have now been included as a Supplementary Information file.

In addition, the affiliation of Dr. Mumtaz Jabeen was incorrect, as she was unaffiliated at the time of this research. Therefore, her listed affiliation,

“Section of Genetics, Department of Zoology, Aligarh Muslim University, Aligarh, 202002, India”

now reads:

“Aligarh, India”

