# Supplementary material for: Therapeutic potential of functionalized siRNA nanoparticles on regression of liver cancer in experimental mice
Source: Sci Rep. 2019 Nov 1;9:15825. doi: 10.1038/s41598-019-52142-4 (PMC6825139; doi:10.1038/s41598-019-52142-4)
Supplement: Supplementary file 1 — Supplementary Information. [file 41598_2022_52142_MOESM1_ESM.docx]

**Therapeutic potential of functionalized siRNA nanoparticles on regression of liver cancer in experimental mice**

**Azmat Ali Khan^1^*, Amer M. Alanazi ^1^, Mumtaz Jabeen, Arun Chauhan^2^ & Mohammad Azam Ansari^3^**

^1^Pharmaceutical Biotechnology Laboratory, Department of Pharmaceutical Chemistry, College of Pharmacy, King Saud University, Riyadh, 11451, Saudi Arabia. ^2^ Department of Neuroimmunology, School of Health and Medicine, University of North Dakota, Grand Forks, ND, USA. ^3^Department of Epidemic Disease Research, Institutes of Research and Medical Consultations (IRMC), Imam Abdulrahman Bin Faisal University, 31441, Dammam, Saudi Arabia.

***Correspondence to:*** *Azmat Ali Khan, E-mail address:* [*azmatbiotech@gmail.com*](mailto:azmatbiotech@gmail.com)

***Keywords:*** siRNA; N-acetylgalactosamine; asialoglycoprotein receptor; hepatocellular carcinoma; nanoconjugate; PEGylated PLGA


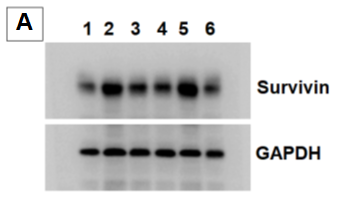


**Figure S1:** The originals of Western blots for Figure 9A.


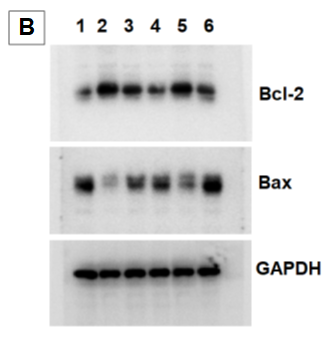


**Figure S2:** The originals of Western blots for Figure 9B.
